# Genetic polymorphisms of ATG5 predict survival and recurrence in patients with early-stage esophageal squamous cell carcinoma

**DOI:** 10.18632/oncotarget.20793

**Published:** 2017-09-08

**Authors:** Pei-Wen Yang, Min-Shu Hsieh, Ya-Han Chang, Pei-Ming Huang, Jang-Ming Lee

**Affiliations:** ^1^ Department of Surgery, National Taiwan University Hospital and National Taiwan University College of Medicine, Taipei, Taiwan; ^2^ Department of Pathology, National Taiwan University Hospital and National Taiwan University College of Medicine, Taipei, Taiwan; ^3^ Graduate Institute of Pathology, College of Medicine, National Taiwan University, Taipei, Taiwan

**Keywords:** esophageal squamous cell carcinoma (ESCC), ATG5, SNP, recurrence

## Abstract

Esophageal squamous cell carcinoma (ESCC) is a deadly disease with high risk of tumor recurrence even among patients with an early pathologic stage of tumor. In the current study, we investigate the association between 20 SNPs of the ATG5 gene and prognosis of patients with early-stage ESCC. A total of 305 patients diagnosed with early-stage ESCC were enrolled in the study and randomly assigned to a training set (n=93) or replication set (n=212). The genotypes of candidate SNPs (single nucleotide polymorphisms) within ATG5 were analyzed and correlated with the prognosis of ESCC patients. We repeatedly demonstrated that 3 SNPs in ATG5, rs1322178, rs3804329, and rs671116, were significantly correlated with the prognosis of patients with early-stage ESCC (HR[95 % CI]=2.01[1.19-3.40], p=0.009 for ATG5: rs1322178; HR[95 % CI]=1.88 [1.08-3.26], p=0.025 for ATG5:rs3804329; HR[95 % CI]=1.73[1.24-2.42], p=0.001 for ATG5:rs671116, in combined group). Both rs1322178 and rs3804329 can predict early distant metastasis of patients. Furthermore, increased expression of ATG5 was observed in ESCC tumor tissue as compared to adjacent normal tissue. Moreover, higher levels of ATG5 expression in both normal and tumor tissues exhibited a trend to correlate with poor prognosis of patients. However, the expression of ATG5 did not correlate with these 3 relevant prognostic SNPs. We concluded that hereditary genetic polymorphisms and gene expression of *ATG5* can serve as prognostic predictors of patients with early-stage ESCC.

## INTRODUCTION

Esophageal cancer is among the major causes of cancer death worldwide [1–2]. It presents mainly as either esophageal squamous cell carcinoma (ESCC) or esophageal adenocarcinoma (EA) in histology [1]. The standard treatment for locally advanced esophageal cancer is neoadjuvant (preoperative) concurrent chemoradiotherapy (CCRT) with or without surgery. Patients who respond well to CCRT are restaged as pathologically early-stage after treatment. A high risk of recurrence has been found even among those diagnosed with a pathologically early-stage of tumor [3–4]. More than 50% of the patients with primary esophageal cancer encounter local-regional recurrence or distant metastases within 2 to 3 years [5–7]. The median survival after recurrence of ESCC is only about 8 months [7].

Autophagy is the “self-eating” molecular machinery involved in the bulk lysosomal degradation of long-lived proteins and organelles, which serves to maintain cellular homeostasis [8–9]. The genes involved in the process of autophagy are termed autophagy-related genes (ATG). Autophagy has been shown to be correlated with tumor formation and progression, and with cancer therapy outcomes [9–11]. The role of autophagy in tumorigenesis is complicated and is likely to be background dependent. A functional autophagy mechanism may be a necessary homeostatic process which removes damaged organelles and thus protects against cancer [12]. However, it may also promote cancer cell survival and growth in response to growth-limiting conditions such as nutrient depletion and hypoxia [13–14]. Autophagic activity has also been demonstrated to correlate with sensitivity to radiation or chemotherapy in treating various cancers [15–17]. Numerous anti-cancer therapies are known to induce autophagy. Targeting autophagy during cancer therapy is, therefore, regarded as a potential approach to improve the clinical outcome of cancer patients [10].

Autophagy has been suggested as a potential mechanism for resistance of ESCC to therapy [18–21]. Induction of autophagy by drug-resistant esophageal cancer cells was found to promote their survival and recovery following treatment with chemotherapeutics [18]. Specific inhibition with siRNA of early autophagy induction targeted to ATG7 and Beclin 1obviously enhanced the effect of 5-FU (5-Fluorouracil) and reduced the recovery of drug-treated esophageal cancer cells [18]. Autophagy inhibition was also observed to contribute to radiation sensitization of ESCC [21]. However, a well-known autophagy inducer, lithium, has been reported to enhance the efficacy of therapeutic agents in esophageal cancer [22].

The ATG5 gene encodes autophagy protein 5 (Atg5), which associates with Atg12 and Atg16 to form an Atg5-Atg12/Atg16 complex that is essential for the formation of autophagosomes during the process of autophagy [10]. ATG5 is thus a possible factor involved in the tumor recurrence in early-stage esophageal cancer, yet it has hardly been investigated.

We thus set out to investigate the association of ATG SNPs (single nucleotide polymorphisms) with the prognosis of early-stage ESCC and found that SNPs at ATG5 gene are significantly associated with the prognosis of early-stage ESCC (Supplementary Table 1). The correlation of ATG5 expression in ESCC tissues and both prognosis and genotype of early-stage ESCC patients were also investigated.

## RESULTS

A total of 305 patients pathologically diagnosed with early-stage (stage 0, I and II) ESCC were enrolled in the study and randomly assigned to a training set (n=93) or replication set (n=212). The distributions of the demographic and clinical characteristics in the total patient group were compared by survival and recurrence status (Table 1). As expected, stage, T-stage and N-stage were strongly associated with both mortality and tumor recurrence (Table 1). Gender was also significantly associated with both survival and disease recurrence (P=0.014 for survival and P=0.001 for recurrence, Table 1). The management of patients, including surgical resection (esophagectomy) and CCRT was also strongly correlated with disease recurrence (P=0.021 for surgical resection and P=0.026 for CCRT treatment).

**Table 1:** Patient characteristics

	Total	Survival		Recurrence			
Variables		Dead	Alive	*p*-value	no recurrence	recurrence	*p*-value
		**201 (65.9)**	**104 (34.1)**		**78 (25.6)**	**227 (74.4)**	
**Age**				0.057			0.281
<40	70	38 (54.3)	32 (45.7)		23 (32.9)	47 (67.1)	
40-60	144	98 (68.1)	46 (31.9)		34 (23.6)	110 (76.4)	
>60	91	65 (71.4)	26 (28.6)		21 (23.1)	70 (76.9)	
**Sex**				**0.014**			**0.001**
Male	278	189 (68.0)	89 (32.0)		64 (23.0)	214 (77.0)	
Female	27	12 (44.4)	15 (55.6)		14 (51.9)	13 (48.1)	
**Stage**				**<0.001**			**<0.001**
0	50	28 (56.0)	22 (44.0)		17 (34.0)	33 (66.0)	
I	112	62 (55.4)	50 (44.6)		40 (35.7)	72 (64.3)	
II	143	111 (77.6)	32 (22.4)		21 (14.7)	122 (85.3)	
**T-stage**				**0.006**			**0.004**
0	69	45 (65.2)	24 (34.8)		19 (27.5)	50 (72.5)	
1	100	54 (54.0)	46 (46.0)		36 (36.0)	64 (64.0)	
2	94	71 (75.5)	23 (24.5)		17 (18.1)	77 (81.9)	
3	41	31 (75.6)	10 (24.4)		5 (12.2)	36 (87.8)	
4	1	0 (0)	1 (100)		1 (100)	0 (0)	
**N-stage**				**0.001**			**0.001**
0	245	152 (62.0)	93 (38.0)		71 (29.0)	174 (71.0)	
1	59	49 (83.1)	10 (16.9)		6 (10.2)	53 (89.8)	
2	1	0 (0)	1 (100)		1 (100)	0 (0)	
**Tumor location**			0.117				0.107
Upper	61	47 (77.0)	14 (23.0)		10 (16.4)	51 (83.6)	
Middle	149	95 (63.8)	54 (36.2)		45 (30.2)	104 (69.8)	
Lower	95	59 (62.1)	36 (37.9)		23 (24.2)	72 (75.8)	
**Operation**				0.173			**0.021**
No	27	21 (77.8)	6 (22.2)		2 (7.4)	25 (92.6)	
Yes	278	180 (64.7)	98 (35.3)		76 (27.3)	202 (72.7)	
**CCRT**				0.051			**0.026**
No	100	57 (57.0)	43 (43.0)		34 (34.0)	66 (66.0)	
Yes	197	139 (70.6)	58 (29.4)		41 (20.8)	156 (79.2)	
CT	1	1 (100.0)	0 (0)		0 (0)	1 (100)	
RT	6	4 (66.7)	2 (33.3)		2 (33.3)	4 (66.7)	
CT+RT	1	0 (0)	1 (100.0)		1 (100.0)	0 (0)	

The genotypes of 20 candidate ATG SNPs were analyzed from the genomic DNA of 93 ESCC patients in the training group. The genotypes of the early-stage ESCC patients were correlated with survival by multivariate Cox regression analysis using dominant (Dom), recessive (Rec) and additive models. Three ATG5 SNPs, rs1322178, rs3804329 and rs671116, were found to be significantly or borderline associated with overall survival of patients by either the dominant, the recessive or the additive model (Supplementary Table 1).

In patients with early-stage ESCC, the genetic variants of ATG5:rs1322178 (HR[95 % CI]=3.60 [1.40-9.26], p=0.008), ATG5:rs3804329 (HR[95 % CI]=3.06[1.13-8.31], p=0.029), and ATG5:rs671116 (HR[95 % CI]=1.95[1.03-3.71], p=0.041, recessive model) were significantly associated with increased risk of death in the training set (Supplementary Table 1 and Table 2). Significant association of ATG5: rs1322178 and ATG5: rs671116 with risk of death was further confirmed in the replication group (HR[95 % CI]=1.99[1.02-3.90], p=0.045 and HR[95 % CI]=1.59 [1.06-2.41], p=0.027 for ATG5: rs1322178 and ATG5: rs671116, respectively, Table 2). The genotypes of ATG5:rs3804329 displayed borderline association with overall survival in the replication group (p=0.064, Table 2). All of these SNPs were significantly correlated with hazard of death in the combined group (HR[95 % CI]=2.01[1.19-3.40], p=0.009 for ATG5:rs1322178; HR[95 % CI]=1.88[1.08-3.26], p=0.025 for ATG5:rs3804329; HR[95 % CI]=1.73[1.24-2.42], p=0.001 for ATG5:rs671116, Table 2). Notably, these ATG5 SNPs can predict early recurrence (i.e., recurrence within 2 years). of early-stage ESCC. Compared to the CC variant, the CT variant of ATG:rs1322178 had a 7.03-fold increased risk of early local recurrence (OR [95 % CI]=7.03 [0.99-49.99], P=0.051, Table 3) and a 4.50-fold increased risk of early distant metastasis (OR [95 % CI]= 4.50 [1.19-17.01], P=0.027, Table 3). Patients carrying the AG genotype of ATG5:rs3804329 also had a 4.5-fold increased hazard of early distant metastasis (OR [95 % CI]= 4.50 [1.19-17.01], P=0.027, Table 3) compared to patients with the AA genotype.

**Table 2:** Association of SNPs in ATG5 gene with mortality of early-stage ESCC patients under multivariate analysis

			Training group		Replication group		Combined group	
SNP	Function	genotype	HRs (95% CI)	p-value	HRs (95% CI)	p-value	HRs (95% CI)	p-value
			n=93		n=212		n=305	
**ATG5: rs1322178**	3’UTR	CC	1		1		1	
		CT	3.60 (1.40-9.26)	**0.008**	1.99 (1.02-3.90)	**0.045**	2.01 (1.19-3.40)	**0.009**
**ATG5: rs3804329**	intron	AA	1		1		1	
		AG	3.06 (1.13-8.31)	**0.029**	1.95 (0.96-3.94)	0.064	1.88 (1.08-3.26)	**0.025**
**ATG5: rs671116**	intron	CC+CT	1		1		1	
		TT	1.95 (1.03-3.71)	**0.041**	1.59 (1.06-2.41)	**0.027**	1.73 (1.24-2.42)	**0.001**

^*^Adjusted for age, gender, stage, surgicalstatus and CCRT**.**

**Table 3:** Association of SNPs in ATG5 gene with early recurrence of early-staged ESCC patients under multivariate analysis

			Early local recurrence			Early distant metastasis	
SNP	Genotype	n	ORs (95% CI)	p-value	n	ORs (95% CI)	p-value
**ATG5: rs1322178**	CC	158	1	0.051	217	1	**0.027**
	CT	6	7.03 (0.99-49.99)		12	4.50 (1.19-17.01)	
**ATG5: rs3804329**	AA	159	1	0.131	217	1	**0.027**
	AG	5	5.23 (0.61-44.72)		12	4.50 (1.19-17.01)	
**ATG5: rs671116**	CC+CT	126	1	0.129	174	1	0.252
	TT	38	2.09 (0.81-5.43)		55	1.50 (0.75-2.98)	

^*^Adjusted for age, gender, stage, surgicalstatus and CCRT.

**Figure 1:**
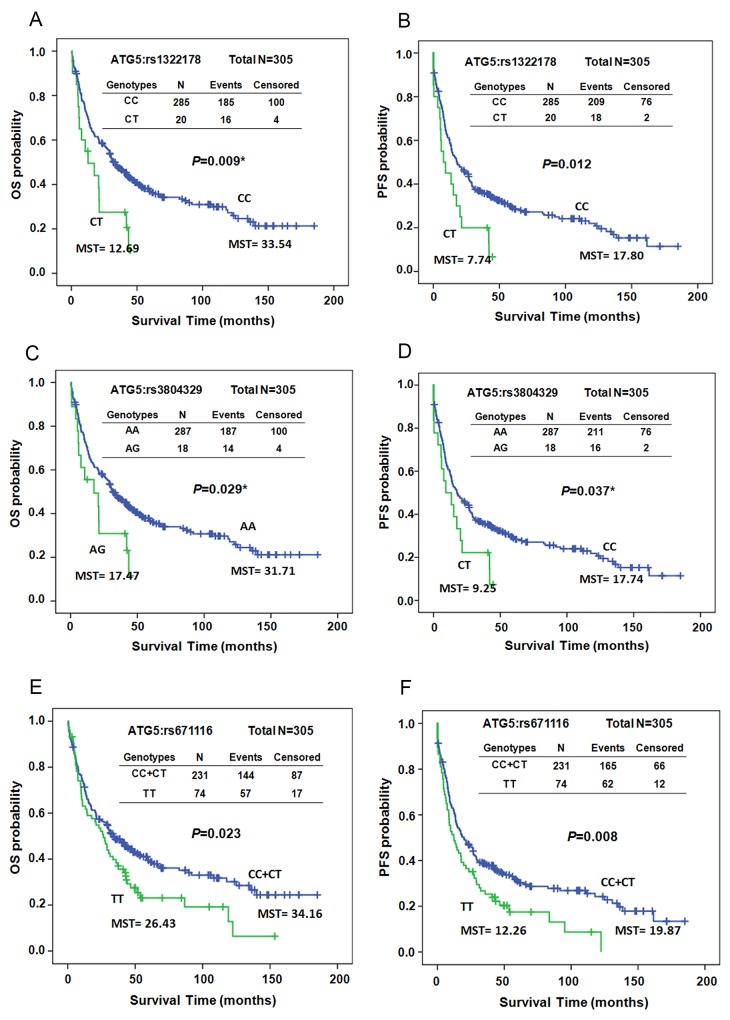
Kaplan–Meier estimates of overall survival (OS, **A, C,** and **E)** or progression-free survival (PFS, **B, D,** and **F)** by the genotypes of ATG5:rs1322178 (A and B), ATG5:rs3804329 (C and D), and ATG5:rs671116 (E and F) in early-stage ESCC patients. MST: median survival time.

The Kaplan–Meier survival curves revealed that both OS and PFS differed significantly between patients with and without the variant allele T of ATG5:rs1322178 in early-stage patients (P=0.009 for OS and P=0.012 for PFS, Figure 1A and 1B). Patients carrying variant genotype CT exhibited decreases in both OS and PFS (mean survival time [MST] 33.54 vs. 12.69 months for OS; MST 17.80 vs. 7.74 months for PFS, Figure 1A and 1B). Both OS and PFS were significantly shorter in patients with the variant allele of ATG5:rs3804329 (MST 31.71 vs. 17.47 months, P=0.029 for OS; MST 17.74 vs. 9.25 months, P= 0.037 for PFS, Figure 1C and 1D). Patients carrying the TT genotype of ATG5:rs671116 also had significantly shorter OS and PFS compared to patients with the CC or CT genotypes (MST 34.16 vs. 26.43 months, P=0.023 for OS; MST 19.87 vs. 12.26 months, P= 0.008 for PFS, Figure 1E and 1F).

**Figure 2:**
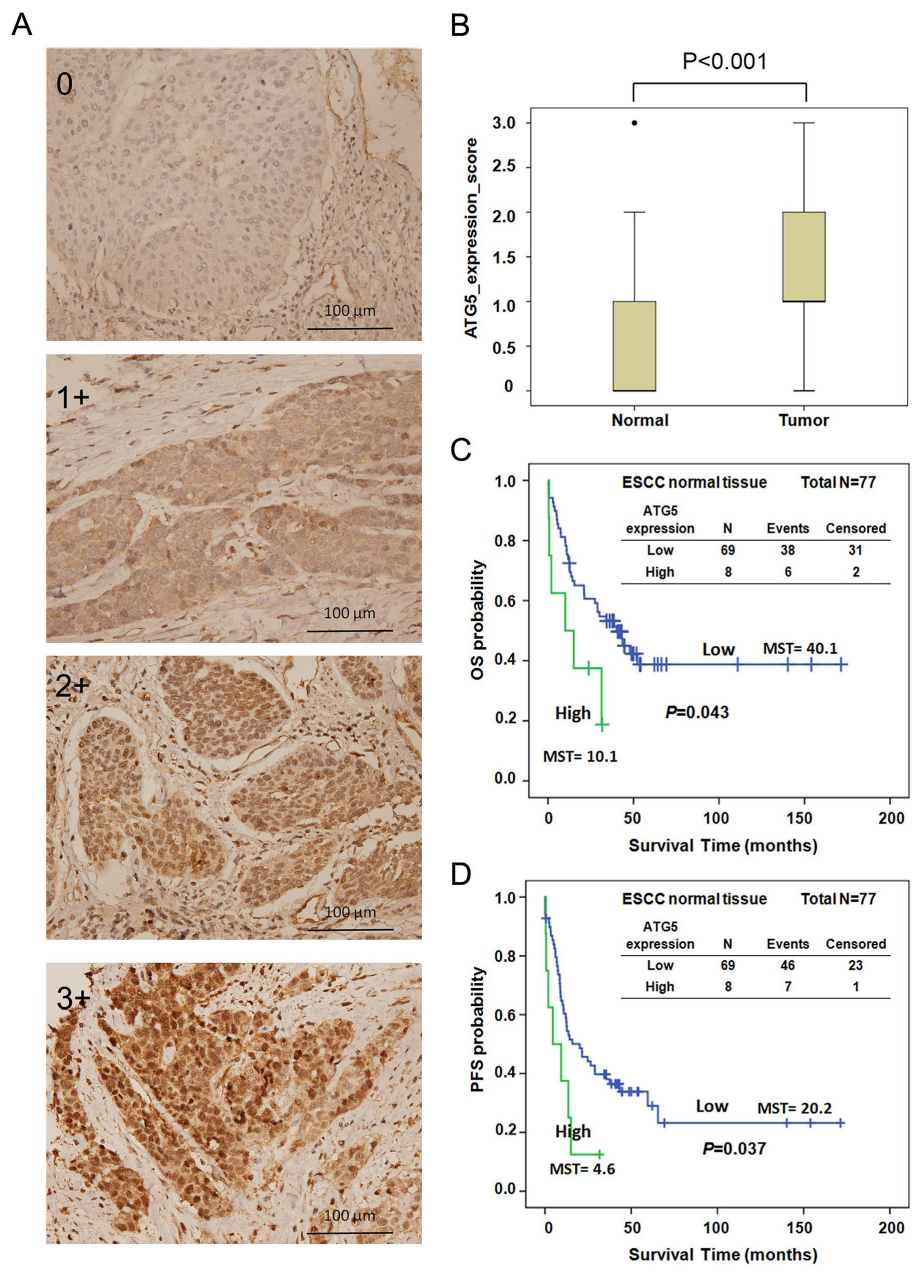
**(A)** ATG5 expression in ESCC tissue was analyzed by IHC and scored as 0, 1+, 2+, and 3+. **(B)** Expression level of ATG5 in adjacent non-cancerous (normal) and early-stage ESCC tissues by IHC. **(C-D)** Kaplan–Meier estimates of OS (C) and PFS (D) by the expression levels of ATG5 (low and high) of adjacent normal tissue from early-stage ESCC patients. MST: median survival time. Low, expression score 0 or 1+; High, expression score 2+ or 3+.

To investigate whether the effect of the ATG5 SNPs on the prognosis of patients was mediated by modulating the expression of ATG5, we analyzed the expression levels of ATG5 in tumor and adjacent normal tissues from patients with early-stage ESCC by IHC. The expression levels were scored as 0+ (no detection), 1+ (low), 2+ (medium), and 3+ (high) (Figure 2A and Supplementary Table 2). Approximately 77.4 % of the ESCC tumor samples tested positive for ATG5. In adjacent normal tissues, ATG5 could be detected in about 48 % of the samples (Supplementary Table 2). The expression of ATG5 was significantly up-regulated in tumor tissues compared to its expression in adjacent normal tissue (Figure 2B, P<0.001, independent t-test). We further categorized the expression level of ATG5 into low (scoring 0+ or 1+) and high (scoring 2+ or 3+) expression groups. High expression of ATG5 in normal tissue was significantly correlated with increased risk of tumor progression compared to low expression (HR [95 % CI]=1.82 [0.99-3.35], P=0.033, Table 4). Patients whose tumor tissue had elevated expression of ATG5 exhibited a trend of higher risk of adverse clinical outcome compared to those with low expression, but without reaching statistical significance (HR [95 % CI]=1.43 [0.87-2.34], P=0.159 for OS, HR [95 % CI]=1.41 [0.88-2.27], P=0.150 for PFS, Table 4). Re-classifying the groups into low (scoring 0), middle (scoring 1 or 2), and high (scoring 3), high ATG5 expression in tumor tissue had a 2.23-fold higher hazard of death compared to low expression (HR [95 % CI]=2.23 [1.06-4.68], P=0.035, Table 4).

**Table 4:** Association of ATG5 expression in both normal and tumorous tissue with overall and progression-free survival of early-staged ESCC

Variables	N	Overall survival	^*^P-value	Progression-free survival	^*^P-value
		HR (95 % CI)		HR (95 % CI)	
**Normal_ATG5 expression**					
Low	69	1		1	
High	8	2.01 (0.82-4.93)	0.126	1.82 (0.99-3.35)	**0.033**
**Tumor_ATG5 expression**					
Low	69	1		1	
High	46	1.43 (0.87-2.34)	0.159	1.41 (0.88-2.27)	0.150
**Tumor_ATG5 expression**					
Low	26	1		1	
Middle	69	1.20 (0.67-2.16)	0.540	0.98 (0.57-1.69)	0.942
High	20	2.23 (1.06-4.68)	**0.035**	1.49 (0.73-3.06)	0.277

^*^Adjusted for age, gender, and stage.

Survival curves were also constructed for OS and PFS by the expression level of ATG5. The median survival time of OS and PFS decreased significantly as ATG5 expression elevated (MST 40.1 vs. 10.1 months, log-rank P=0.043 for OS, Figure 2C; MST 20.1 vs. 4.6 months, log-rank P=0.037 for PFS, Figure 2D). However, neither OS nor PFS differed significantly in patients with different expression levels of ATG5 in tumor tissue (data not shown). The expression level of ATG5 was further evaluated with the genetic polymorphisms of ATG5. Unexpectedly, the expression level of ATG5 in both normal and tumor tissue did not exhibit significant correlation with the genotypes of ATG5:rs1322178, ATG5:rs3804329, or ATG5:rs671116 (Supplementary Table 3).

## DISCUSSION

What role the SNPs of the ATGs might play in ESCC prognosis has not been investigated. In the current study, we demonstrated that 3 SNPs in ATG5, rs1322178, rs3804329, and rs671116, significantly correlated with the prognosis of patients with early-stage ESCC (Table 2 and Figure 1). Both ATG5:rs1322178 and ATG5: rs3804329 can predict early distant metastasis of early-stage ESCC (Table 3). Meanwhile, ATG5 expression was significantly higher in ESCC tumor tissues than in adjacent normal tissue (Figure 2A-2B). Higher expression of ATG5 in both normal and tumor esophageal tissues had a trend to correlate with adverse clinical outcome of patients (Table 4 and Figure 2C-2D). However, the ATG5 SNPs did not have an associated effect on the expression of ATG5 (Supplementary Table 3).

ATG5: rs1322178 is located within the 3’ untranslated region (3’-UTR), whereas rs3804329 and rs671116 are in the intron region of the ATG5 gene. Studies of these 3 ATG5 SNPs are rare, and their function is hardly known. A previous study found that these 3 SNPs were all located in the same haplotype block of strong LD (linkage disequilibrium) and had no significant association with childhood asthma [23]. Therefore, these SNPs may display similar associations with early ESCC prognosis due to their close proximity.

Genetic variation in 3’UTR has been shown to often correlate with mRNA stability mediated by post-translation modification or microRNA interaction [24]. We observed alteration of the nucleotide from wildtype C to variant T of ATG5: rs1322175, supporting the notion that these nucleotides might be targeted by different mature microRNAs by sequence alignment. We thus hypothesized thatrs1322178 may regulate ATG5 expression by modulating RNA stability in ESCC. Unexpectedly, there was no significant correlation in esophageal tissue between ATG5 protein expression and genotype of any these 3 SNPs despite the fact thatATG5 expression exhibited prognostic correlation in ESCC. Since these ATG5 SNPs did not exert an obvious effect on the regulation of ATG5 expression, we infer that these 3 ATG5 SNPs did not directly affect the prognosis of ESCC by regulating ATG5 expression. There might be some other SNP within the exon region of ATG5 and located in the same haplotype block with these 3 SNPs that influences the function of ATG5 by a structural change rather than a change in expression of ATG5.

ATG5 is a cellular factor with an ambiguous role in malignant transformation. It has been known to promote Ras-induced cell transformation since autophagy was demonstrated to be involved in the oncogenic event [25–26]. In addition to the function of autophagy, ATG5 plays a role as a pro-apoptotic molecule after being cleaved at residue Thr 193 by calpain (a calcium dependent protease) indicating a molecular switch between autophagy and apoptosis [27–28]. ATG5 gene knockdown by small interference RNA (siRNA) has also been reported to enhance starvation-induced cell death [27, 29].

Both up-regulation and down-regulation of ATG5 have been demonstrated in various tumor tissues. Down-regulation of ATG5 has been found in colorectal cancer and early-stage cutaneous melanoma tissue compared to their normal counter parts [30–31]. Partial loss of ATG5 has also been observed in gastric and hepatocellular carcinomas [32]. Notably, elevated ATG5 was correlated with lympho vascular invasion even though ATG5 was decreased in colorectal cancer [30]. Increased expression of ATG5 has been observed in oral squamous cell carcinoma (OSCC) and prostate cancers [33–34]. ATG5 expression was found associated with tumor grade, tumor size, clinical stage and lymph node metastasis and clinical outcome in OSCC [33]. Meanwhile, increased expression of ATG5 was also significantly correlated with adverse prognosis and chemo-resistance in gastric cancer [35].

The expression of ATG5 in ESCC has never been previously reported. Our current study demonstrates that ATG5 expression was markedly increased in early-stage ESCC tissue compared to adjacent non-tumorous tissue even though we did not observe correlation of ATG5 expression with pathologic characteristics (data not shown). We thus reasonably suggest that ATG5 might be involved in malignant transformation of esophageal squamous cells. Even though K-Ras mutation in esophageal cancer is rare [36], upstream factors such as EGFR (epidermal growth factor receptor) are frequently over-expressed in esophageal cancer [37]. Therefore, ATG5 might participate in autophagy and promote esophageal cell transformation mediated by EGFR-Ras signaling. Moreover, ATG5 might also be induced by hypoxia, a cellular stress known to induce autophagy [38], since HIF-1α (hypoxia-inducible factor-1 alpha) was found to express in ESCC cells [39–40].

A high level of ATG5 expression in normal tissue is, even more so than in cancer tissue, significantly associated with an adverse clinical outcome in early-stage ESCC. Over-expression of ATG5 in ESCC cells is possibly correlated with autophagic events which induce drug resistance and tumor growth to lead to poor prognosis. Adjacent normal cells expressing ATG5 might induce cell apoptosis to prevent tumorigenesis. However, the apoptosis of surrounding normal cells might also promote tumor growth based on the concept of cell competition [41], and lead to cancer progression. Blocking apoptosis of the adjacent normal cells has been hypothesized to be a novel pathway to prevent tumor growth [42].

In conclusion, our study demonstrates for the first time the prognostic relevance of the genetic polymorphisms and expression of ATG5 in patients with ESCC. These results reveal a novel functional mechanism involved in tumor progression of ESCC and provide a novel biomarker for predicting the clinical outcome of patients with ESCC. A limitation of our study is that no elucidation of the underlying mechanism regarding the prognostic function of ATG5 was provided, a goal well worth pursuing in further research.

## MATERIALS AND METHODS

### Study population

This retrospective study, investigating a total of 305 patients with early-stage (pathological stage (stage 0, I or II) ESCC collected in the surgical department of National Taiwan University Hospital (NTUH) from 2000 to 2013, was approved by the research ethics committee (201205090RIC). Patients histologically confirmed with early-stage (stage 0, I or II) primary ESCC, or those with locally advanced ESCC who were restaged as early-stage after CCRT, were included. Pregnant women, pediatric patients, and those unable to give informed consent were excluded. Cisplatin-based neoadjuvant concurrent chemoradiation therapy (CCRT) was administered to patients with locally advanced ESCC. Esophagectomy was performed on those patients with resectable disease status and acceptable surgical risk after CCRT. Information regarding demographics, tumor location, treatment protocols, recurrence status, and TNM stage according to the AJCC 7^th^ edition [43], was obtained through medical chart review. Overall survival (OS) duration was defined as the interval between initial diagnosis of the disease (in patients who did not undergo surgery) or surgery for the disease and mortality of the patient. Progression-free survival (PFS) was defined as the interval between diagnosis of or surgery for the disease and detection of local recurrence, disease progression of the tumor, or death. Recurrence within 2 years after surgery (or initial diagnosis in patients without surgery) were considered as early recurrence, whether recurrence was local only or included distant metastasis.

### DNA extraction

The buffy coat was isolated from a 5 ml whole blood sample collected from each patient before treatment and was stored in a -80 ^o^ C freezer. Genomic DNA was extracted from the buffy coat with the QIAamp DNA Mini Kit (Qiagen, Hamburg Germany) following the manufacturer’s instructions.

### Genotyping

Based on the results of previous studies, 20 candidate SNPs were selected, which consisted of 1 SNP at autophagy related 3 (ATG3), 6 SNPs at autophagy related 5 (ATG5), 9 SNPs at autophagy related 7 (ATG7), 3 SNPs at autophagy related 16-like 1 (ATG16L1), and 1 SNP at beclin 1 (BECN1) (Supplementary Table 1) [23, 44–47]. The SNP genotyping was performed with the Sequenom MassARRAY platform and iPLEX gold chemistry following manufacturer’s instructions (Sequenom, San Diego, CA, USA). Briefly, specific PCR primer and extension primer sequences for multiplex PCR reaction were designed using the Assay Designer software package (v.4.0). After multiplex PCR, the residual deoxynucleotides were deactivated by incubation with 0.3 U of shrimp alkaline phosphatase followed by single base extension reaction. Seven μl of purified reaction mixture was loaded onto a matrix pad of a SpectroCHIP (Sequenom) and analyzed by MassARRAY Analyzer 4. Genotypes were called by cluster analysis using MassARRAY TYPER 4.0 software and call rates higher than 80 % were accepted. Artifact data were removed manually. Data integrity and accuracy were confirmed by repeated measures.

### Immunohistochemistry (IHC)

Formalin-fixed paraffin-embedded (FFPE) blocks of ESCC patient tissue collected during surgical intervention were obtained from the department of pathology in National Taiwan University Hospital. Cancer and normal esophagus FFPE sections were dewaxed and rehydrated. Details of the IHC protocol were described in a previous study [48]. The primary antibody used was a rabbit polyclonal antibody against ATG5 (1:200, NB110-53818, Novus Biologicals).

### Statistical analysis

Patient characteristics and ATG5 expression among the subgroups with different genotypes of ATG SNPs were compared using a Pearson’s χ^2^ test or Fisher’s exact test. The hazard ratios (HRs) of death and disease progression were obtained from multivariate Cox regression analysis adjusted for potential significant covariates. The odds ratios (ORs) obtained by logistic regression were used to describe correlations between genotypes and early recurrence or protein expression of ATG5.

The correlations between genotypes or ATG5 expression and OS or PFS were obtained using the Kaplan–Meier method and compared using the log-rank test. The ATG5 protein expression levels of esophageal tissues among normal and cancer tissue were analyzed by box-plot and independent t-test. All statistical analyses were conducted with SPSS 17.0 for Windows (SPSS Institute, Chicago, IL, USA). A *p*-value ≤ 0.05 was considered statistically significant.

## SUPPLEMENTARY MATERIALS TABLES




