# Supplementary material for: Genetic polymorphisms of ATG5 predict survival and recurrence in patients with early-stage esophageal squamous cell carcinoma
Source: Oncotarget. 2017 Sep 8;8(53):91494–504. doi: 10.18632/oncotarget.20793 (PMC5710940; doi:10.18632/oncotarget.20793)
Supplement: Supplementary file 1 [file oncotarget-08-91494-s001.pdf]

Genetic polymorphisms of ATG5 predict survival and recurrence in patients with early-stage esophageal squamous cell carcinoma

SUPPLEMENTARY MATERIALS

Supplementary Table 1: Association of ATG-related SNPs with overall survival of early-staged ESCC patients in training group under multivariate analysis(recessive, dominant, and additive models).

See Supplementary File 1

Supplementary Table 2: Expression of ATG5 in normal and tumor esophageal tissues

	ATG5 in normal tissue		ATG5 in tumor tissue	
Score	N	Percent	N	Percent
**0**	40	51.9	26	22.6
**1+**	29	37.3	43	37.4
**2+**	7	9.1	26	22.6
**3+**	1	1.3	20	17.4
**total**	77	100	115	100

Supplementary Table 3: Association of ATG5 expression in both normal and tumorous tissue with the genotypes of ATG5 SNPs in patients with early-staged ESCC

ATG5 expression		Normal tissue			Tumor tissue		
SNP	Genotype	Low	High	p-value	Low	High	p-value
**rs1322178**	**CC**	16 (88.9)	8 (11.9)	0.248	62 (59.6)	42 (40.4)	1.000
	**CT**	8 (100.0)	0 (0)		7 (63.6)	4 (36.4)	
**rs3804329**	**AA**	61 (88.4)	8 (11.6)	0.590	63 (59.4)	43 (40.6)	0.739
	**AG**	8 (100.0)	0 (0)		6 (66.7)	3 (33.3)	
**rs671116**	**CC+CT**	53 (89.8)	6 (10.2)	1.000	54 (58.7)	38 (41.3)	0.568
	**TT**	16 (88.9)	2 (11.1)		15 (65.2)	8 (34.8)	
