# Supplementary material for: Genetic polymorphisms of ATG5 predict survival and recurrence in patients with early-stage esophageal squamous cell carcinoma
Source: Oncotarget. 2017 Sep 8;8(53):91494–504. doi: 10.18632/oncotarget.20793 (PMC5710940; doi:10.18632/oncotarget.20793)
Supplement: Supplementary file 2 [file oncotarget-08-91494-s002.docx]

**Supplementary Table 1: Association of ATG-related SNPs with overall survival of early-staged ESCC patients in training group under multivariate analysis(recessive, dominant, and additive models)**

| GENE | SNP | type | genotypes | Recessive model | | | genotypes | Dominant model | | | Additive model | |  |
| --- | --- | --- | --- | --- | --- | --- | --- | --- | --- | --- | --- | --- | --- |
|  |  |  |  | N | Adjusted HRs  (95% CI) | p-value |  | N | Adjusted HRs  (95% CI) | p-value | Adjusted HRs  (95% CI) | p-value |  |
| ATG3 | rs11547153 | nonsense | CC | 93 | - | - | CC | 93 | - | - | - | - |  |
| ATG5 | rs12201458 | intron | CC | 93 | - | - | CC | 93 | - | - | - | - |  |
| ATG5 | rs1322178 | 3'UTR | CC | 87 | 1 |  | CC | 87 | 1 |  |  |  |  |
|  |  |  | CT | 6 | 3.60 (1.40-9.26) | **0.008** | CT | 6 | 3.60 (14.0-9.26) | **0.008** | 3.60 (14.0-9.26) | **0.008** |  |
| ATG5 | rs3804329 | intron | AA | 88 | 1 |  | AA | 88 | 1 |  |  |  |  |
|  |  |  | AG | 5 | 3.06 (1.13-8.31) | **0.029** | AG | 5 | 3.06 (1.13-8.31) | **0.029** | 3.06 (1.13-8.31) | **0.029** |  |
| ATG5 | rs510432 | 5'UTR | GG+GA | 76 | 1 |  | GG | 36 | 1 |  |  |  |  |
|  |  |  | AA | 17 | 1.54 (0.77-3.05) | 0.221 | GA+AA | 57 | 0.91 (0.54-1.54) | 0.728 | 1.08 (0.74-1.57) | 0.699 |  |
| ATG5 | rs573775 | intron | CC+CT | 80 | 1 |  | CC | 36 | 1 |  |  |  |  |
|  |  |  | TT | 13 | 1.26 (0.63-2.56) | 0.514 | CT+TT | 57 | 0.66 (0.39-1.11) | 0.113 | 0.85 (0.57-1.26) | 0.413 |  |
| ATG5 | rs671116 | intron | CC+CT | 70 | 1 |  | CC | 26 | 1 |  |  |  |  |
|  |  |  | TT | 23 | 1.95 (1.03-3.71) | **0.041** | CT+TT | 67 | 0.89 (0.51-1.55) | 0.676 | 1.20 (0.80-1.78) | 0.381 |  |
| ATG7 | rs1499082 | intron | AA+AG | 85 | 1 |  | AA | 54 | 1 |  |  |  |  |
|  |  |  | GG | 8 | 0.73 (0.28-1.94) | 0.527 | AG+GG | 39 | 0.96 (0.58-1.60) | 0.874 | 0.93 (0.63-1.36) | 0.695 |  |
| ATG7 | rs1802496 | missense | CC | 93 | - | - | CC | 93 | - | - | - | - |  |
| ATG7 | rs2606742 | intron | TT | 92 | 1 |  | TT | 92 | 1 |  |  |  |  |
|  |  |  | CT | 1 | 4.14 (0.34-51.11) | 0.268 | CT | 1 | 4.14 (0.34-51.11) | 0.268 | 4.14 (0.34-51.11) | 0.268 |  |
| ATG7 | rs2606750 | intron | GG+GA | 85 | 1 |  | GG | 46 | 1 |  |  |  |  |
|  |  |  | AA | 8 | 0.81 (0.32-2.03) | 0.650 | GA+AA | 47 | 1.21 (0.74-1.99) | 0.453 | 1.08 (0.74-1.57) | 0.705 |  |
| ATG7 | rs346078 | intron | GG | 86 | 1 |  | GG | 86 | 1 |  |  |  |  |
|  |  |  | CC | 7 | 1.27 (0.52-3.08) | 0.601 | CC | 7 | 1.27 (0.52-3.08) | 0.601 | 1.27 (0.52-3.08) | 0.601 |  |
| ATG7 | rs35807939 | missense | GG | 93 | - | - | GG | 93 | - | - | - | - |  |
| ATG7 | rs36117895 | missense | TT | 93 | - | - | TT | 93 | - | - | - | - |  |
| ATG7 | rs3856794 | intron | CC+CG | 86 | 1 |  | CC | 54 | 1 |  |  |  |  |
|  |  |  | GG | 7 | 6.36 (0.39-103.59) | 0.194 | CG+GG | 39 | 2.03 (0.36-11.35) | 0.423 | 2.09 (0.62-7.07) | 0.238 |  |
| ATG7 | rs4684787 | intron | CC+CT | 83 | 1 |  | CC | 39 | 1 |  |  |  |  |
|  |  |  | TT | 10 | 1.85 (0.33-10.54) | 0.487 | CT+TT | 54 | 1.12 (0.68-1.85) | 0.647 | 1.02 (0.47-2.21) | 0.956 |  |
| ATG16L1 | rs1866878 | missense | CC | 93 | - | - | CC | 93 | - | - | - | - |  |
| ATG16L1 | rs2241879 | intron | CC+CT | 82 | 1 |  | CC | 49 | 1 |  |  |  |  |
|  |  |  | TT | 11 | 1.02 (0.49-2.13) | 0.961 | CT+TT | 44 | 1.34 (0.83-2.18) | 0.237 | 1.16 (0.83-1.63) | 0.386 |  |
| ATG16L1 | rs2241880 | missense | TT+TC | 82 | 1 |  | TT | 48 | 1 |  |  |  |  |
|  |  |  | CC | 11 | 1.02 (0.49-2.13) | 0.961 | TC+CC | 45 | 1.29 (0.80-2.09) | 0.304 | 1.14 (0.81-1.60) | 0.447 |  |
| Beclin-1 | rs34128114 | missense | CC | 93 | - | - | CC | 93 | - | - | - | - |  |

*Adjusted for age, gender, stage, surgical status and CCRT
